# Cytokinin‐induced promotion of root meristem size in the fern *Azolla* supports a shoot‐like origin of euphyllophyte roots

**DOI:** 10.1111/nph.13630

**Published:** 2015-09-11

**Authors:** Jan de Vries, Angela Melanie Fischer, Mayo Roettger, Sophie Rommel, Henriette Schluepmann, Andrea Bräutigam, Annelie Carlsbecker, Sven Bernhard Gould

**Affiliations:** ^1^ Molecular EvolutionHeinrich‐Heine‐University DüsseldorfUniversitätsstr. 140225DüsseldorfGermany; ^2^ Population GeneticsHeinrich‐Heine‐University DüsseldorfUniversitätsstr. 140225DüsseldorfGermany; ^3^ Molecular Plant PhysiologyUtrecht UniversityPadualaan 83584CH Utrechtthe Netherlands; ^4^ Plant BiochemistryHeinrich‐Heine‐University DüsseldorfUniversitätsstr. 140225DüsseldorfGermany; ^5^ Department of Organismal Biology, Physiological BotanyUppsala BioCenterLinnean Centre for Plant BiologyUppsala UniversityUlls väg 24ESE‐756 51UppsalaSweden

**Keywords:** apical development, auxin, cytokinin, fern, meristem regulation, RNAseq, root development, root evolution

## Abstract

- The phytohormones cytokinin and auxin orchestrate the root meristem development in angiosperms by determining embryonic bipolarity. Ferns, having the most basal euphyllophyte root, form neither bipolar embryos nor permanent embryonic primary roots but rather an adventitious root system. This raises the questions of how auxin and cytokinin govern fern root system architecture and whether this can tell us something about the origin of that root.
- Using *Azolla filiculoides*, we characterized the influence of IAA and zeatin on adventitious fern root meristems and vasculature by Nomarski microscopy. Simultaneously, RNAseq analyses, yielding 36 091 contigs, were used to uncover how the phytohormones affect root tip gene expression.
- We show that auxin restricts *Azolla* root meristem development, while cytokinin promotes it; it is the opposite effect of what is observed in *Arabidopsis*. Global gene expression profiling uncovered 145 genes significantly regulated by cytokinin or auxin, including cell wall modulators, cell division regulators and lateral root formation coordinators.
- Our data illuminate both evolution and development of fern roots. Promotion of meristem size through cytokinin supports the idea that root meristems of euphyllophytes evolved from shoot meristems. The foundation of these roots was laid in a postembryonically branching shoot system.

## Introduction

Roots are a key innovation of vascular plants. They probably arose twice, once in the fern lineage and once in lycopods (Pires & Dolan, 2012). Hence, the emergence of the complex sporophyte body was accompanied by the emergence of roots (Graham *et al*., 2000; Doyle, 2013). Phylogenetically early‐branching bryophytes also form root‐like rhizoids. Regardles of whether they are uni‐ or multicellular (Jones & Dolan, 2012), rhizoids are elementary structures that grow through tip growth in the same manner as root hairs (Menand *et al*., 2007). Gametophytes of ferns form rhizoids, too (Banks, 1999), but in contrast to the bryophytes, their life cycle is dominated by the sporophytic stage (as in angiosperms), in which they build proper roots (Gunning *et al*., 1978; Banks, 1999). During the evolutionary transition to a sporophyte‐dominated plant life cycle, regulators of gametophyte development were recruited for sporophyte development (Menand *et al*., 2007; Frank & Scanlon, 2015). Gametophyte and sporophyte development in basal land plants share control mechanisms (Landberg *et al*., 2013; Bennett *et al*., 2014; Viaene *et al*., 2014), but we know little about complex roots of basal vascular plants beyond that.

Two major apical meristems determine complex plant growth: the shoot apical meristem (SAM) and the root apical meristem (RAM). Exploring the root's origin is therefore exploring the origin of the RAM. Apical development through a SAM‐like structure precedes the emergence of the RAM (Graham *et al*., 2000; Prigge & Bezanilla, 2010) and regulation of the SAM and RAM show striking similarity (Stahl & Simon, 2010). It has therefore been speculated that the initial RAM evolved from the SAM (Stahl & Simon, 2010), a transition that must have happened in the early vascular plants. To understand this transition, we explored the molecular regulation of the fern RAM in comparison to that of angiosperms.

The *Arabidopsis* RAM has become the model system to study angiosperm meristem function (Petricka *et al*., 2012). While *Arabidopsis* forms a taproot system (allorhizy) that is dominated by the embryonic primary root, other plants such as grasses develop a fibrous root system (secondary homorhizy). Regardless of the mature root system architecture, angiosperms establish a primary root (and RAM) during early embryogenesis, serving angiosperm seedlings of both allorhizic and homorhizic species (Bellini *et al*., 2014). Angiosperm embryos establish the apical–basal polarity already at the two‐cell stage, a process orchestrated by polar auxin flow (Friml *et al*., 2003; Petrasek & Friml, 2009). Next to auxin, root identity in embryogenesis is determined by master regulators such as *WUSCHEL RELATED HOMEOBOX* (*WOX*) genes (Haecker *et al*., 2004; Schlereth *et al*., 2010; Radoeva & Weijers, 2014). Angiosperm roots develop longitudinally in three different zones: the meristematic zone (MZ), from which all root tissues arise, the elongation zone (EZ), where the cells reach their final size, and the differentiation zone (DZ), where elongated cells reach full maturation according to their respective fate (Dolan *et al*., 1993).

All tissues of the root originate from the stem cell niche's (SCN) initials (Petricka *et al*., 2012) that line the quiescent centre (QC), which itself remains mitotically inactive but maintains the meristematic activity of its surroundings (Clowes, 1954; van den Berg *et al*., 1997). Activity and establishment of the SCN are orchestrated through a few key regulators such as PLETHORA (PLT; Aida *et al*., 2004), SHORTROOT (SHR) and SCARECROW (SCR; Scheres *et al*., 1995; Sabatini *et al*., 2003). PLT is a further key determinant of the meristem size (Galinha *et al*., 2007). Activity and size of the RAM are regulated by the antagonists auxin and cytokinin (Blilou *et al*., 2005; Dello Ioio *et al*., 2007, 2008). While auxin promotes the MZ size through cell proliferation (Blilou *et al*., 2005), cytokinin restricts it by promoting earlier cell differentiation (Dello Ioio *et al*., 2007, 2008). The Aux/IAA auxin signalling repressor (Tian & Reed, 1999) SHORT HYPOCOTYL 2 (SHY2) (that is also targeted by the cytokinin signalling components RESPONSE REGULATOR 1 (ARR1) and ARR12; Sakai *et al*., 2001; Ishida *et al*., 2008; Moubayidin *et al*., 2010) determines the position of the transition zone (TZ; marking the border between MZ and EZ) and therefore the size of the MZ (Dello Ioio *et al*., 2008; Moubayidin *et al*., 2010; Perilli *et al*., 2012). The same appears true for the formation of adventitious roots in angiosperms, where auxin promotes and cytokinin hinders adventitious rooting (Werner *et al*., 2003; Gutierrez *et al*., 2012; Bellini *et al*., 2014).

Fern embryos do not develop in a bipolar manner (Hou & Hill, 2002). The embryonic RAM is only briefly present and the permanent fern roots arise adventitiously from a shoot, a process termed primary homorhizy (Goebel, 1930; Schneider, 2013) that is clearly distinguished from (secondary) homorhizic angiosperms with long‐living embryonic roots (Hou & Hill, 2002; Bellini *et al*., 2014). Instead of bearing a QC‐centred SCN, fern roots have a single root apical cell (RAC) that gives rise to root tissues (Gunning *et al*., 1978; Hou & Blancaflor, 2009). Our knowledge about the molecular mechanisms underlying the function of the fern RAC is limited, but Nardmann & Werr (2012) could show that *WUSCHEL*‐related transcription factors act in the RAC of *Ceratopteris*, demonstrating them to be ancient master regulators of plant development that radiated even before the angiosperm–gymnosperm split (Hedman *et al*., 2013). Studies on *Ceratopteris* showed that auxin did not alter lateral root formation, but inhibited parent root growth (Hou *et al*., 2004). Yet the molecular framework that underpins fern meristem regulation remained hidden. We used *Azolla filiculoides*, famous for its unique symbiosis with a nitrogen‐fixing cyanobacterium (Rai *et al*., 2000; Carrapiço, 2010), to characterize how the key developmental regulators auxin and cytokinin influence fern root development. While the longitudinal organization of the *Azolla* root resembles that of angiosperms, we found that the phytohormonal meristem regulation through auxin and cytokinin is reversed. Our results shed light on the evolutionary origin of the RAM in euphyllophytes.

## Materials and Methods

### *Azolla* culture and phytohormone treatment

*Azolla filiculoides* Lam. was cultivated in floating culture in a beaker containing 250 ml filtered water (pH 7.0) under 450 μmol quanta m^−2^ s^−1^ 16 h 24°C : 8 h 20°C, light: dark, day : night, and 75% relative humidity. For phenotypic analysis, 2 d after root removal (hereafter defined as 2 dpc) roots were treated with 2.7 μl EtOH (mock), 0.1 μM IAA (Carl‐Roth; IAA, Karlsruhe, Germany) and 0.5 μM *trans*‐Zeatin (Sigma‐Aldrich; CK) for 24 h.

### Phenotypic analysis

For differential interference contrast (DIC) analyses, roots were mounted in a chloral hydrate solution (5 : 2 : 1, chloral hydrate : glycerol : H_2_O) and bleached (i.e. tissues were cleared) for several days. Images were taken on a Nikon Ti Eclipse microscope (Nikon DS‐Qi1Mc camera). Root lengths (entire 3 dpc root) were determined via dissection microscopy (Nikon SMZ 745T; Nikon DS‐Ri1 camera). Images were processed using the NIS‐Elements BR 4.20.00 software (Nikon, Tokyo, Japan) and Adobe Photoshop and Illustrator CS6. Statistical analysis was performed using R 3.0.2 (R Core Development Team 2013), normality was tested using a Shapiro–Wilk test (Shapiro & Wilk, 1965) and, accordingly, a Mann–Whitney *U‐*test (Mann & Whitney, 1947) was performed. Amyloplasts were stained using 5% Lugol's iodine and pictures were taken on a Zeiss Axiophat microscope with an AxioCam ICc 5 camera, using the ZEN 2012 software (Zeiss). Dissection micrographs were generated using a SteREO Discovery V8 (Zeiss) microscopy with a AxioCAM ICc 5 and processed using the ZEN 2012 software (Zeiss).

### Global gene expression analysis

For RNAseq analysis, 66 hpc roots were treated as described above but for 6 h; 3–4 mm root tips were collected and extracted using the Spectrum^™^ Plant Total RNA Kit (Sigma) according to the manufacturer's instructions. RNA was extracted for each treatment in triplicate (*n *≥* *100 root tips each), always at the same time of the day. RNA quality was assessed using a formamide gel and shipped on dry ice to BGI Tech Solutions (Hong Kong), where RNA quality assessment by BioAnalyzer (Agilent Technologies, Waldbronn, Germany), library preparation using the TruSeq kit (Illumina, San Diego, CA, USA), and 100 bp paired‐end sequencing via the Illumina HiSeq2000 system were performed. After removal of low‐quality reads, we obtained a total of 270 866 324 reads (Supporting Information Table S1). The Transcriptome Shotgun Assembly project has been deposited at DDBJ/EMBL/GenBank under the accession GBTV00000000 and the  National Center for Biotechnology Information (NCBI) Single Read Archive (PRJNA264391). The version described in this paper is the first version, GBTV01000000.

Read quality was assessed using Fastqc v.0.10.1 (FASTQC 2012) and trimmed using Trimmomatic 0.32 (Bolger *et al*., 2014; settings: ILLUMINACLIP:TruSeq3‐PE.fa:2:30:10; HEADCROP:10; TRAILING:3; SLIDINGWINDOW:4:20; MINLEN:36). All reads were paired‐end assembled using Trinity r20131110 (Grabherr *et al*., 2011; Haas *et al*., 2013), resulting in 153 400 contigs (≥ 300 bp). Reads were mapped onto the contigs using the CLC Genomics Workbench 7 (CLC Bio). Contigs were annotated based on a BLASTx approach (Altschul *et al*., 1997) against the TAIR 10 peptide release (Lamesch *et al*., 2011); based on the false discovery rate, the *e‐*value cutoff was set to 10^−7^. Filtering for contigs with ≥ 150 mapped reads yielded 36 091 contigs (Table S2). Potential contamination was omitted by retaining only contigs that had best BLASTx hits (vs RefSeq; Pruitt *et al*., 2012) to streptophytes.

Gene onthology (GO) term analysis was performed based on the *Arabidopsis* annotations (*e*‐ value < 10^−7^) using the Gene Ontology enRIchment anaLysis and visuaLizAtion tool (GOrilla;* P *< 10^−3^) (Eden *et al*., 2009).

Differential regulation and statistical analysis were performed based on negative binomial probability distribution and Benjamini–Hochberg correction using edgeR (Robinson *et al*., 2010). Wordles were generated using wordle.net. Phytohormone signalling pathways were manually curated based on *Arabidopsis* homologues (Liscum & Reed, 2002; Lamesch *et al*., 2011; Brenner & Schmüllig, 2012). Quantitative reverse transcription polymerase chain reaction (qRT‐PCR) was performed using the Power SYBR^™^ Green kit and a StepOnePlus^™^ (Applied Biosystems) system and analysed according to Pfaffl (2001). *Af*TufA (AzfiRT00021) and *Af*CAM5 (AzfiRT00154) were selected as reference genes based on their steady expression (confirmed during qPCR analysis on equal amounts of RNA) in the RNAseq data (see Table S3 for primer sequences).

### Protein family matrix

Protein data were extracted from current genome releases (The Arabidopsis Genome Initiative, 2000; Merchant *et al*., 2007; Rensing *et al*., 2008; Schnable *et al*., 2009; The International Brachypodium Initiative, 2010; Banks *et al*., 2011; The Potato Genome Sequencing Consortium, 2011; Nystedt *et al*., 2013; Amborella Genome Project, 2013) from NCBI and Joint Genome Institute (JGI) and the longest open reading frames (start to stop; ≥ 100 aa; *in silico* translated using Emboss v.6.6.0; Rice *et al*., 2000) of RNAseq and expressed sequence tag (EST) data from *Spirogyra pratensis* (Timme & Delwiche, 2010), *Marchantia polymorpha* (Nagai *et al*., 1999), *Ceratopteris richardii* (Bushart *et al*., 2013; same filtering as for the *Azolla* RNAseq dataset) and *Pteridium aquilinum* (Der *et al*., 2011). Orthologous protein families were determined using a bidirectional best hit (Tatusov *et al*., 1997) all‐against‐all BLASTp v.2.2.29+ approach (*e‐*value ≤ 10^−10^; global sequence identity ≥ 30% calculated by Emboss v.6.6.0 Needle; Rice *et al*., 2000) and clustered using the Markov Cluster Algorithm (MCL) (Enright *et al*., 2002). Results were displayed in a Matlab R2014a (MathWorks, Natick, MA, USA)‐generated matrix.

### Hierarchical clustering of expansin expression and phylogeny

An expansin dataset was generated based on best‐BLASTx hits against the *Azolla* root transcriptome and sequences from Sampedro & Cosgrove (2005), Li *et al*. (2002), the NCBI and the Arabidopsis Information Resource **(**TAIR; Table S4). Protein sequences were generated using the sequence manipulation suite (Stothard, 2000), aligned using Multiple Alignment using Fast Fourier Transform (Mafft) v.7.127b L‐INS‐I (Katoh & Standley, 2013) and Mega5.2.2 (Tamura *et al*., 2011) and modified to include only the conserved middle region of the protein sequences. Sequences containing only parts of the conserved region were removed. A maximum‐likelihood phylogeny was computed using WAG + G + I (best‐fit substitution model determined by Mega5.2.2; five discrete gamma categories, 1000 bootstrap replicates, partial deletion site coverage cutoff: 99%). Sequences were annotated based on best BLAST hits to *Arabidopsis* expansins, unless annotation was given. Hierarchical clustering on the normalized expression data was performed by using log_2_(FC) data. Data for 3 h CK‐ and IAA‐treated *Arabidopsis thaliana* seedlings were extracted from The Bio‐Analytic Resource for Plant Biology (BAR) (Toufighi *et al*., 2005; Winter *et al*., 2007). Clusters were generated using the CLC main workbench 7.0 (CLCbio, Qiagen) and Euclidean distance measures with single linkage.

## Results

### *Azolla* has adventitiously emerging shoot‐borne roots

**Figure 1:**
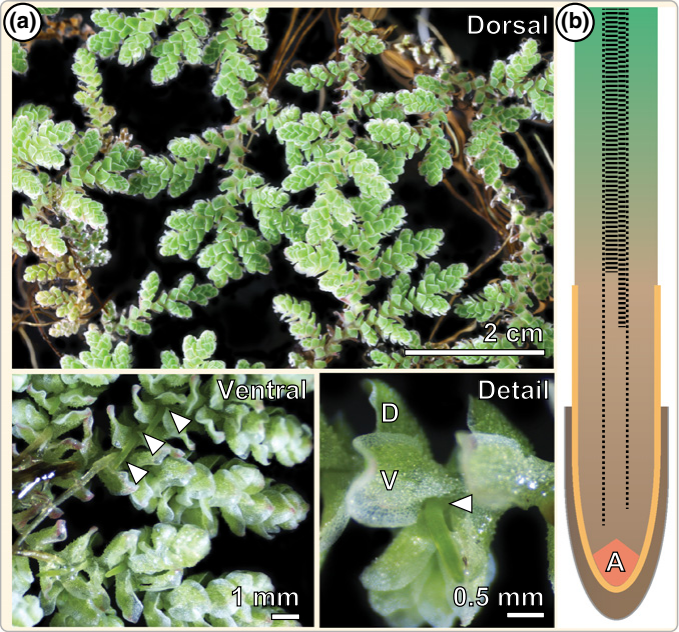
Emergence of shoot‐borne *Azolla filiculoides* roots. (a) Stereoscopic micrographs of *Azolla filiculoides* sporophytes in floating culture, showing a dorsal (upper panel), ventral (lower left panel) and detailed view (lower right panel). Arrows mark the shoot‐borne, adventitious emergence of roots. The detailed view shows the nodes containing a dorsal (D) and a ventral (V) leaf lobe. (b) Schematic drawing of the *Azolla* root that highlights the outer root cap (brown), the inner root cap (yellow), the apical cell (A), and differentiation of the xylem strands in dashed lines (outer thin lines represent protoxylem, and inner thick lines represent metaxylem).

**Figure 2:**
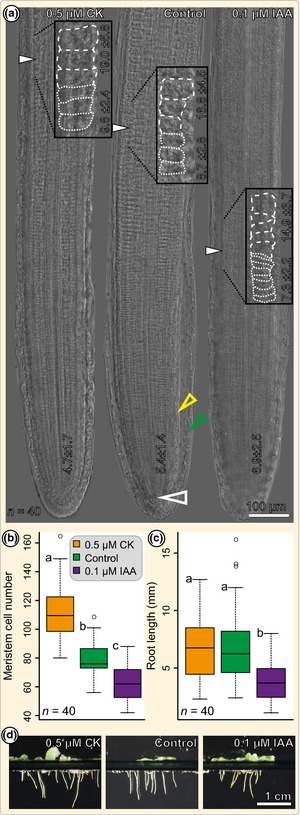
*Azolla* root meristem cell number is elevated upon cytokinin and decreased upon auxin treatment. (a) Nomarski interference contrast micrographs of *Azolla filiculoides* roots treated with solvent (control), 0.5 μM *trans*‐zeatin (CK) and 0.1 μM IAA. Open arrows mark the root apical cell (RAC) and closed arrows mark the end of the outer cortex meristematic zone (MZ). Inserts show an enlarged view of the outer cortex transition zone (TZ), marking the end of the MZ and giving the average length of the last three cells of the MZ and the first three cells of the elongation zone (EZ) in µm (± SD; cells are retraced by dashed lines). Numbers at the apex give the average length of the first 10 outer cortex cells in μm (± SD). The green arrowhead marks the outer root cap, the yellow arrowhead marks the inner root cap. (b) Quantification of the outer cortex MZ cell number in mock (control)‐, 0.5 μM CK‐ and 0.1 μM IAA‐treated 3 d post‐cut (dpc) roots; significance groups a–c (*P *<* *0.001) were determined using Mann‐Whitney *U*‐statistics. (c) Quantification of the root length in mock (control)‐, 0.5 μM CK‐ and 0.1 μM IAA‐treated 3 dpc roots; significance groups a and b (*P *<* *0.001) were determined using Mann–Whitney *U*‐statistics. Box‐plots in (b) and (c) display the interquartile range (IQR; 50 ± 25%) of the data; horizontal lines in each box mark the median (50%), whiskers extend to the furthest data points within the 1.5 × IQR range, and circles mark outliers. (d) Photographs of 3 dpc *A. filiculoides* sporophytes in floating culture upon mock (control), 0.5 μM CK and 0.1 μM IAA treatment. All presented data points are derived from evaluation of at least 40 roots per treatment (*n* = 40).

*Azolla* roots emerge adventitiously as shoot‐borne roots (cf. Groff & Kaplan, 1988) from a node that contains a ventral and a dorsal leaf lobe (Fig. 1a). Roots of angiosperms can be longitudinally separated into the DZ, EZ and MZ (Dolan *et al*., 1993), a partitioning that we also used for the roots of *Azolla*. The largest and uppermost segment of the *Azolla* root is made up by the DZ, where the cells reached their final size, the xylem is differentiated and root hairs are prominent. The EZ begins with cells that are approximately twice as long as those of the MZ (from 8.7 ± 2.5 μm of the three outer cortex cells before the doubling to 16.8 ± 4.5 μm of the three outer cortex cells after the doubling, marking the TZ; Fig. 2a). At the tip lies the MZ, housing the RAC. Trichoblasts, from which the root hairs later emerge, are already distinguishable in the MZ as a result of their triangular appearance; proper differentiation of the root hairs occurs at the end of the MZ and subtending the inner root cap (Fig. S1). *Azolla* roots bear an outer (Fig. 2a green arrowhead) and an inner root cap (Figs 1b, 2a yellow arrowhead). The outer root cap ends approximately at the TZ, while the inner root cap ends approximately at the transition between EZ and DZ. Potassium iodide (KI) staining revealed no statocytes where one would expect a columella, but instead several basipetal amyloplasts lining the stele (Fig. S2). In contrast to the fern *Ceratopteris* (Hou *et al*., 2004; Hou & Blancaflor, 2009), *Azolla* does not have lateral roots.

### Size of the *Azolla* root MZ is increased by cytokinin and decreased by auxin

Auxin and cytokinin are key regulators of the MZ's activity in angiosperms and thus we analysed their effect on the *Azolla* root's MZ. To make sure that only meristems of the same age and developmental stage were analysed, roots were first removed. At 1 dpc, *Azolla* roots had replaced the removed roots but showed nonuniformal phenotypes. We used the distinct TZ to evaluate the *Azolla* MZ (starting from first emergence, cells of the outer cortex file were counted until they had doubled in size) and determined that 3 dpc roots showed the steadiest MZ size (Fig. S3), which were thereafter used for all analyses. Ferns were kept in floating culture for 2 dpc and transferred to a new culture containing either solvent (mock treatment) or 0.1 μM IAA or 0.5 μM *trans*‐zeatin (CK) for 24 h. The concentrations were chosen based on previous studies on meristem size in *Arabidopsis* (Růžička *et al*., 2009; Dello Ioio *et al*., 2012; Moubayidin *et al*., 2013), as well as preliminary experiments determining the minimal concentrations resulting in clear phenotypes. Mock‐treated roots (3 dpc) had an MZ cell number of 77 ± 12 (Fig. 2a,b). Exogenous 0.1 μM IAA application led to roots with a significant reduction (*P *<* *0.001) in MZ size (62 ± 12; Fig. 2a,b). By contrast, application of 0.5 μM CK increased the MZ size (111 ± 19, *P *<* *0.001; Fig. 2a,b). Application of the phytohormones also influenced the lengths of the MZ cells (Fig. 2a). In mock‐treated roots, the first 10 outer cortex cells had an average length of 5.4 ± 1.4 μm; IAA treatment increased this to 6.9 ± 1.4 μm (*P *<* *0.001), while CK treatment decreased cell size to 4.7 ± 1.7 μm (*P *<* *0.001). Control 3 dpc roots were 6.81 ± 3.1 mm in length. While 0.5 μM CK – although changing the meristem cell number – led to no significant change in root length (6.81 ± 2.7 mm; *n* = 40), 0.1 μM IAA resulted in a significant decrease (*P *<* *0.001), to 3.73 ± 1.6 mm (Fig. 2c,d).

### Auxin and cytokinin affect xylem formation

**Figure 3:**
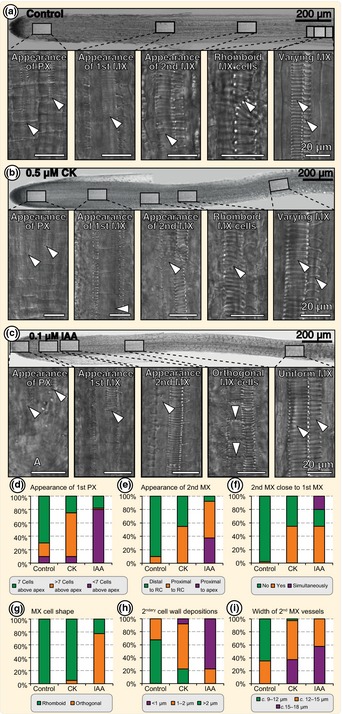
*Azolla* root xylem development upon cytokinin and auxin treatments. (a–c) Nomarski interference contrast micrographs of *Azolla filiculoides* roots treated with solvent (control), 0.5 μM *trans*‐zeatin (CK) and 0.1 μM IAA; blow‐ups of marked positions along the root show details of the xylem phenotypes, marked with arrowheads. A, root apical cell; PX, protoxylem; MX, metaxylem. (d–i) Quantification of observed alterations in the xylem phenotype after control, 0.5 μM CK and 0.1 μM IAA treatment. Data points are derived from evaluation of at least 40 roots per experiment (*n* = 40). Root cap (RC).

Auxin and cytokinin are also key regulators of root vasculature in *Arabidopsis* (Bishopp *et al*., 2011) and we therefore analysed their influence on xylem morphology under the same conditions as applied earlier (*n* = 40). *Azolla* roots bear two narrow protoxylem and two wide metaxylem strands (Fig. 1b). The protoxylem is *c*. 4 μm in diameter, progressing from scalariform ornamentation to annular ornamentation (Fig. 3). Protoxylem differentiates, on average, seven stele cells above the apex. One protoxylem strand differentiates before the other (approx. two cells), and differentiating either earlier on IAA or later on CK treatment (Fig. 3d). Metaxylem shows a scalariform ornamentation. First metaxylem strands differentiate as wide cell files (*c*. 8 μm in diameter) more tipwards, and second metaxylem strands differentiate as even wider cell files (*c*. 13 μm in diameter) more shootwards, than the rim of the inner root cap (Fig. 1b). Upon either phytohormone treatment, both metaxylem strands differentiated more towards the tip than in the untreated roots. The relative appearance of the second compared with the first metaxylem strand was also altered such that both strands appeared to start closer to each other (CK and IAA), or even simultaneously (IAA; Fig. 3f). This was accompanied by a change in the perforation plate angle, resulting in orthogonal instead of rhomboid‐shaped metaxylem cells (Fig. 3g). In addition, IAA caused the secondary cell wall deposition leading to the scalariform ornamentation being closer to each other (Fig. 3h). Both phytohormes resulted in a widening of the second metaxylem strand (Fig. 3i). Although varying marginally in size along the root, the second metaxylem strand was always wider than the first.

### Embryophyte developmental regulators of the *Azolla* root

**Figure 4:**
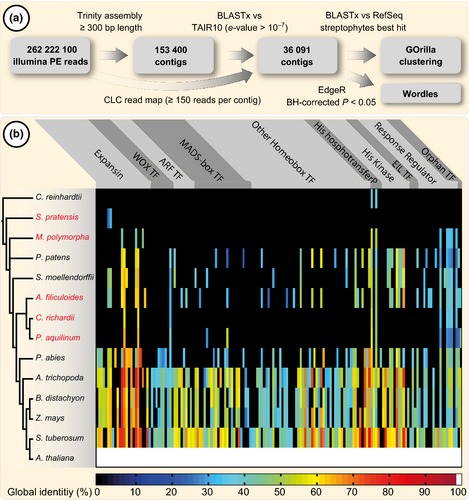
RNAseq of *Azolla* root tips and identification of orthologues to known developmental regulators across 14 species of Chloroplastida. (a) Workflow of the RNAseq analysis of *Azolla filiculoides* root tips starting with the filtered reads obtained by illumina paired‐end (PE) sequencing followed by assembly using the Trinity pipeline, annotation and filtering via BLASTx and read mapping using the CLC workbench. For downstream analyses (protein clustering, expression wordles of significantly regulated genes or gene ontology (GO) term enrichment via GO rilla), only contigs that had their best BLASTx hit to streptophytes were used. (b) Protein data were extracted from nine genomes (black species names) and five RNAseq and expressed sequence tag (EST) libraries (red species names), and clustered; *Chlamydomonas reinhardtii* serves as an outgroup. Clusters were generated from all‐against‐all bidirectional best BLASTp hits. Those clusters were screened for 353 *Arabidopsis thaliana* proteins from 10 gene families (top row) and 161 clusters that contained at least one other species were extracted. The global identity of the identified homologues is displayed as a gradient colour. Note the high number of identified homologues in the *A. filiculoides* root tip transcriptome. The phylogeny on the left is based on the National Center for Biotechnology Information taxonomy database.

Plants colonized land at least 470 million yr ago (Gensel, 2008) and many regulators that shape plant bodies are ancient (Pires & Dolan, 2012). To understand which growth regulators support fern root development, > 270 million paired‐end reads of mRNA isolated from 3‐mm‐long *A. filiculoides* root tips – and under three different conditions (see later) – were sequenced. The *de novo* transcriptome data were assembled into 190 000 contigs, then filtered for those that were supported by ≥ 150 reads and an assembled length of ≥ 300 bp, resulting in 36 091 contigs (Fig. 4a; Table S1). To test for the depth and breadth of key developmental regulators among *Azolla* transcripts, we clustered all proteins derived from available genome, RNAseq and EST data (≥ 100 amino acids long) of a variety of streptophytes (Fig. 4) and *Chlamydomonas reinhardtii* as an outgroup using a bidirectional BLASTp approach. Screening this dataset for clusters that contained orthologues to 353 known *A. thaliana* developmental denominators resulted in 161 clusters that contained at least one orthologue from another species, 40 of which contained homologues expressed in the *A. filiculoides* root. In comparison, proteins from the genomes of *Selaginella moellendorffii* and *Picea abies* were detected in 36 and 58 clusters, respectively, and proteins from the transcriptomes of *C. richardii* and *Pteridium aquilinum* were detected in only 10 and 14 clusters, respectively. Thus, sequence depth of our *de novo* transcriptome was sufficient to detect developmental master regulators, such as homologues of WOX13 (AzfiRT13282, AzfiRT23190, AzfiRT23355; *e‐*value < 10^−47^), bearing the characteristic ancestral NVYNWFQN peptide sequence at the second turn of the recognition helix (Nardmann & Werr, 2012). Key denominators of true root meristems known from angiosperms, such as three homologues of *PLT* (AzfiRT20391, AzfiRT31772, AzfiRT33493; *e‐*value < 10^−95^), were identified (Table S5), accounting for the readout of the meristem regulon. But does differential expression of these key regulators translate into the different phenotypes observed upon phytohormone treatment?

**Figure 5:**
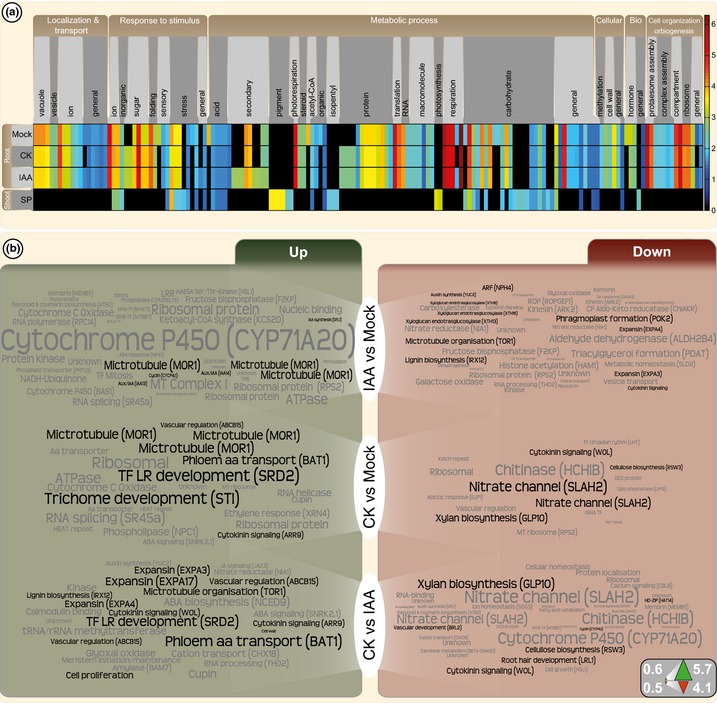
Gene expression changes in the *Azolla* root tip after auxin and cytokinin treatments. (a) Gene ontology (GO) enrichment analysis (*P *< 10^−3^) based on the *Arabidopsis* annotation (*e*‐value < 10^−7^) of the top 2000 expressed genes in 3 d post‐cut (dpc) roots 6 h after treatment with solvent (mock), 0.1 μM IAA and 0.5 μM *trans*‐zeatin (CK) and in the shoots. The fold enrichment of the GO terms is depicted as a colour gradient and GO terms were manually sorted into umbrella categories (top). (b) Significantly differentially regulated (*P *<* *0.05) homologues (*e*‐value < 10^−7^) to *Arabidopsis* expressed in 0.3 mm of the root tip of *Azolla filiculoides*, represented as a word cloud. The bigger the word, the stronger the differential regulation comparing 0.1 μM IAA with mock (IAA vs mock), 0.5 μM CK with mock, and 0.5 μM CK with 0.1 μM IAA treatment. Words in black are of special interest with regard to the main text. Homologues are annotated based on *A. thaliana*; if applicable, the *A. thaliana* gene symbol is provided in brackets. Up‐regulated homologues are shown on the left, down‐regulated homologues on the right. Word sizes are based on log_2_(fold change); see the key in the right corner. For all individual values, see Supporting Information Table S6.

To connect the phenotypic observations to molecular mechanism, RNA was sequenced in triplicate from 3 mm root tips treated for 6 h with 0.1 μM IAA, 0.5 μM CK or solvent (mock). The 6 h treatment was chosen to detect the established readout over initial signalling components of the phytohormone response (Goda *et al*., 2004). Observed trends in expression were confirmed by two‐step qRT‐PCR (Fig. S4). To our knowledge, this is the first global gene expression profiling on a fern root, so we first ran a GO term enrichment analysis (*P *< 10^−3^) on the top 2000 highest expressed genes from all root datasets and compared this with sequence data available from the *Azolla* sporophyte (Brouwer *et al*., 2014) based on homology to *Arabidopsis* proteins (*e‐*value < 10^−7^). The most evident difference is the expression of photosynthesis and pigment metabolism GO terms, detected only in the photosynthesising sporophyte system (Fig. 5a; Table S6). The root expression profiles were enriched for various kinds of transport GO terms, including multifaceted ion transport. Intriguingly, photorespiratory genes were highly expressed in the *Azolla* root, a phenomenon that was recently discussed for angiosperm roots as well (Nunes‐Nesi *et al*., 2014).

Using a ranked GO term enrichment analysis highlighted the differential responses towards IAA and CK (Fig. S5). GO terms for RNA biosynthesis were prominent, indicating that both treatments triggered strong and specific transcriptional responses by the fern. Performing a ranked comparison of genes up‐ and down‐regulated by CK in comparison to IAA highlighted the antagonistic action of the phytohormones. Not only were GO terms for regulation of meristem growth up‐regulated upon CK, but so too were cell cycle regulation and inositol metabolism, which are well‐known growth regulators (Stevenson *et al*., 2000). Importantly, no GO terms associated with stress responses were highlighted. Hence, the reduction of meristem size observed upon auxin treatment is governed by developmental control and not stress.

### Auxin and cytokinin differentially shape *Azolla* roots through cell wall modification

To detect key players and candidate genes for future studies involved in governing *Azolla* root development upon IAA and CK, we pursued a top‐down approach. Screening among contigs with homology to nuclear land plant genes, and which were significantly regulated (Benjamini–Hochberg corrected *P*‐value < 0.05), identified several homologues (*e*‐value < 10^−7^) to *Arabidopsis* growth regulators (Fig. 5b; Table S6). As indicated by the GO terms (Fig. S5) in particular, ‘information processing’ was up‐regulated upon treatment. This included RNA processing such as the RNA splicing SR45 homologue (*e*‐value 1.74 × 10^−09^) and ribosomal proteins in general. Notable was the 54‐fold up‐regulation of a *CYTOCHROME 450 71A20* (*CYP71A20*) homologue, whose orthologue in *A. thaliana* is down‐regulated upon auxin treatment (Goda *et al*., 2004).

**Figure 6:**
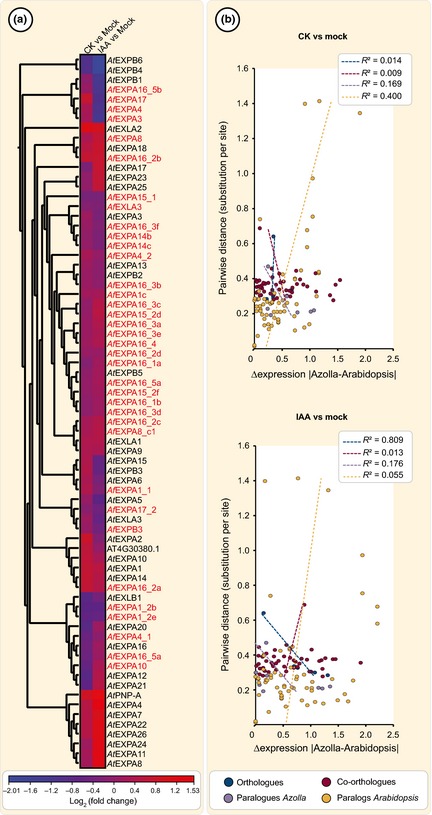
Comparison of *Azolla filiculoides* and *Arabidopsis thaliana* expansin expression patterns in relation to their relatedness. (a) Relative expression of the expansin gene family after *trans*‐zeatin (CK) vs mock treatment (left column) and IAA vs mock treatment (right column) in *A. thaliana* seedlings and *A. filiculoides* roots (in red letters) was clustered hierarchically using Euclidean distance measures and single linkage. Expansin expression is shown in red for up und blue for down and was calculated as log_2_(fold change). Both species‐specific and mixed clusters were obtained, but clusters rarely formed among evolutionarily related expansins. To confirm this relatedness of expansins to each other, a substitution rate per site, estimated with JTT + G + I with five discrete gamma categories (pairwise differences), was correlated to the absolute value of difference in expression between each two expansin genes (Δexpression). (b) No correlation between the pairwise differences and the absolute value of expression differences was observed in any tested phylogenetic category (purple and yellow, paralogues; blue, orthologues; red, co‐orthologues) under CK vs mock treatment. Under IAA vs mock treatment, only a negative correlation for orthologous expansin genes was observed, indicating that, sequence‐wise, highly similar orthologous genes are used differently in ferns and dicots.

Both phytohormone treatments resulted in pronounced expression changes of the cell wall‐modifying expansins (e.g. EXPA4 CK vs mock, 0.3‐fold up in contrast to 2.1‐fold down in IAA vs mock; *P *<* *0.05). The α‐expansins, in particular, were enriched among *Azolla* root transcripts, whereas only one β‐expansin and one α‐expansin‐like were detected. Conserved regions of *Azolla* expansins (Fig. S6b) match well with those of higher embryophytes (Li *et al*., 2002; Sampedro & Cosgrove, 2005), including the α‐ and β‐specific insertions (Li *et al*., 2002). Phylogenetic analysis using 93 protein sequences from 10 embryophytes show a clear distinction between the α‐ and β‐expansins, the expansin‐like sequences clustered with the β‐expansins (Fig. S6). By contrast, other expansin phylogenies (Sampedro & Cosgrove, 2005), expansin sequences of *Azolla filiculoides*,* Physcomitrella patens*,* Marsilea quadrifolia* and *Regnellidium diphyllum* formed lower plant‐specific clades instead of being equally dispersed throughout the α‐sequences. We hierarchically clustered normalized expression data of α‐expansins upon auxin and cytokinin treatment in *A. thaliana* and *A. filiculoides*. Four of the 10 clusters obtained were species‐specific, and only one for *Azolla* (Fig. 6a). The remaining six clusters were dominated by one species, four out of six by *A. filiculoides*. To analyse putative subfunctionalization after species‐specific duplications, we identified potential orthologues, co‐orthologues and paralogues in *A. filiculoides* and *A. thaliana* and estimated pairwise distances within those groups using OrthoMCL (Li *et al*., 2003). In agreement with our phylogenetic analysis (Fig. S6) we obtained only a few orthologues and a larger group of co‐orthologues. When plotting the pairwise distances of each pair against its absolute expression differences, there was little correlation (Fig. 6b). All in all, the data support the conclusion that, based on a common expansin set in all land plants, ferns – perhaps also mosses – have advanced their expansin repertoire, governing a differential use.

Other cell wall modifiers were specifically regulated by the phytohormones as well. A homologue of the cellulose biosynthesis‐associated gene *RADIAL SWELLING3* (*RSW3*;* e*‐value 0) was down‐regulated significantly (*P *<* *0.05), when comparing CK with IAA treatment (3.6‐fold down), and *Arabidopsis rsw3* mutants display a severe root phenotype (Baskin *et al*., 1992; Burn *et al*., 2002). An *IRX12* (IRREGULAR XYLEM 12, an enzyme involved in lignin biosynthesis) homologue (*e*‐value 1.74 × 10^−166^) was significantly (*P *<* *0.05) down‐regulated upon IAA (2.6‐fold) and only marginally down‐regulated upon CK. *Arabidopsis irx12* mutants have strong alterations in xylem formation, displaying collapsing vessel elements (Brown *et al*., 2005), and regulation of *Azolla IRX12* homologues might therefore be correlated with the observed xylem phenotypes.

Key players for the correct xylem cell differentiation are cellulose deposition‐guiding microtubules (Oda & Fukuda, 2012). The expression of several *MICROTUBULE ORGANISATION 1* homologues (*MOR1*;* e*‐values < 1.18 × 10^−107^) increased upon IAA and CK treatment (4.4‐, 4.1‐ and 3.9‐fold up and 4.1‐, 3.8‐ and 3.7‐fold up, respectively; Fig. 5b). A *TORTIFOLIA1* homologue (*TOR1*;* e*‐value 1.02 × 10^−24^) that mediates microtubule‐directed cell wall development (Buschmann *et al*., 2004) was significantly down‐regulated by auxin (2.8‐fold down; *P *<* *0.05). Gene expression changes associated with vascular function were also detected. This included regulation of the ABC transporter ABCB15 (AzfiRT08887; *e*‐value 0; 1.9‐fold down upon IAA treatment) that is expressed in developing vasculature and associated with auxin transport and secondary cell wall development (Kaneda *et al*., 2011) and the phloem amino acid transporter BAT1 homologue (AzfiRT35714; *e*‐value 0), which was 3.7‐fold up‐regulated upon CK treatment (*P *<* *0.05). Intriguingly, nitrate transport‐associated homologues were up to eightfold down‐regulated upon CK treatment (SLAH2; AzfiRT18705, AzfiRT19565), which could be explained by the link between CK biosynthesis and nitrogen availability (Takei *et al*., 2004). IAA and CK, therefore, shape the root of *Azolla* radially and longitudinally by the differential regulation of cell wall‐loosening and ‐reinforcing enzymes.

### Auxin and cytokinin activate specific signalling components in *Azolla* roots

**Figure 7:**
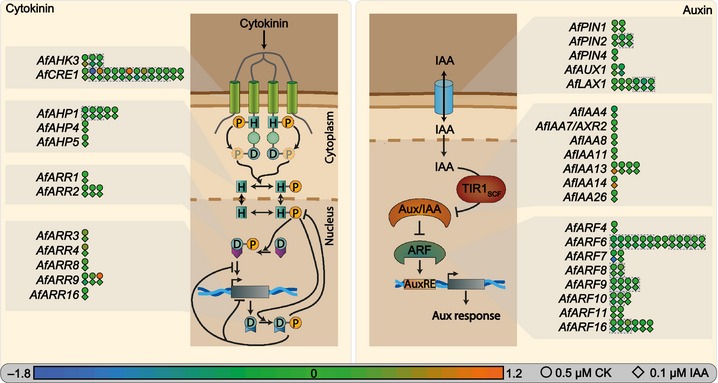
Cytokinin and auxin trigger specific components of their signalling pathways in the *Azolla* root tip. The figure shows the number of detected *Arabidopsis thaliana* cytokinin (left) and auxin (right) signalling pathway components expressed in 0.3 mm root tips of *A. filiculoides*, based on homology (*e*‐value < 10^−7^). Differential expression of each contig is represented by a circle (response to 0.5 μM *trans*‐zeatin, CK) and a rectangle (response to 0.1 μM IAA) which are coloured in a gradient that labels log_2_(fold change) from orange (1.2) to green (0) to blue (−1.8). Although many homologues for the canonical auxin signalling pathway were detected, no homologue of the SCF^TIR^ ^1^ complex could be identified. Grey boxes with dotted lines encase potential isoforms. For all individual values, see Supporting Information Table S6. Aux, auxin.

Root meristems are subject to a tight regulation by auxin and cytokinin. *CYTOKININ RESPONSE 1* (*CRE1*) is a key component in cytokinin signalling (Inoue *et al*., 2001; Fig. 7; Table S6) and *Arabidopsis cre1* mutants have severe root phenotypes (Mähönen *et al*., 2000; Ueguchi *et al*., 2001). Three homologues of *CRE1* (*e*‐values 2.51 × 10^−72^, 4.13 × 10^−39^ and 3.82 × 10^−39^) were found to be regulated upon CK treatment, two up‐ (1.8‐ and 2.0‐fold), one down‐regulated (3.4‐fold; *P *<* *0.05). A total of 14 contigs with homology to *CRE1* (*e‐*values < 10^−14^) were detected in total, only seven of which are predicted to represent isoforms. No other cytokinin signalling component showed a strong response, except for an A‐type *RESPONSE REGULATOR 9* (*ARR9*) homologue (AzfiRT12218; *e*‐value 4.33 × 10^−50^; Figs 5, 7) that was up‐regulated 2.2‐fold (*P *<* *0.001) upon CK treatment.

Expression of three known auxin signalling factors was significantly altered upon auxin treatment (Figs 5, 7). Two *Aux/IAA* homologues were detected, grouping with the B‐group *Aux/IAA* proteins of *Arabidopsis* (Fig. S7; Remington *et al*., 2004) that are known for their importance in primary and lateral root development. One *Azolla Aux/IAA* homologue showed its highest identity to *IAA13* (AzfiRT01636; *e* value 6.64 × 10^−35^; up‐regulated almost two‐fold upon auxin treatment), known to be important for root initiation during embryogenesis and later expressed in the stele (Weijers *et al*., 2005). Besides its phylogenetic placement in the B‐group, the other homologue showed highest identity to *IAA14* (Solitary root; AzfiRT00243; *e* value 1.84 × 10^−35^; up‐regulated almost two‐fold upon auxin treatment). IAA14 is a known regulator of lateral root formation and *Arabidopsis iaa14* mutants do not generate any lateral roots (Fukaki *et  al*., 2002). Finally, a transcript of an *AUXIN RESPONSE FACTOR* was found to be down‐regulated upon IAA treatment, showing highest identity to *ARF7* that is known to be a crucial regulator of lateral root formation (Okushima *et al*., 2007). Also a homologue of the transcription factor *SHOOT REDIFFERENTIATION DEFECTIVE 2* (*SRD2*;* e* value 1.40 × 10^−10^) was found to be up‐regulated upon CK treatment (5.4‐fold; *P *<* *0.05) and *Arabidopsis srd2* mutants are known to have impaired lateral and primary root growth (Ohtani & Sugiyama, 2005). Thus, while several components of the CK and IAA signalling pathway are expressed by the *Azolla* root, only specific factors showed a strong reaction after 6 h of the respective phytohormone treatment, many of which are associated with lateral root formation in *Arabidopsis*.

## Discussion

Understanding meristem regulation is key to understanding plant development. The RAM of *Arabidopsis* has become a priced model to uncover the basic principles of angiosperm development (Petricka *et al*., 2012), but our knowledge about the evolutionary earlier fern root development is rather scarce (Hou & Blancaflor, 2009). Our results highlight the fundamental differences in the control of the *Azolla* RAM by the key regulators cytokinin and auxin.

### Auxin, cytokinin and the reciprocal regulation of the root system in *Azolla*

Similar to *Arabidopsis* (Dolan *et al*., 1993), we found that *Azolla* has a DZ, EZ and MZ. While auxin is known to increase *Arabidopsis* MZ cell number (Blilou *et al*., 2005), cytokinin is known to reduce it (Dello Ioio *et al*., 2007). Similar applies to *Arabidopsis*’ adventitious roots, which one can assume constitute the more obvious analogue to the *Azolla* root. Auxin is a known inductor and promoter of adventitious roots, while cytokinin acts antagonistically (Bellini *et al*., 2014). Surprisingly, application of IAA and CK to the *Azolla* adventitious roots resulted in the opposite effect. Root growth inhibition through auxin is well known in the fern *Ceratopteris* (Hou *et al*., 2004) and also in *Arabidopsis*, where it acts on cell elongation (Rahman *et al*., 2007). Yet the exogenous auxin naphthaleneacetic acid (NAA; acting in a similar manner to IAA on root meristems; Rahman *et al*., 2007) increases MZ cell number in the latter and shows inhibitory effects only at very high concentrations (Růžička *et al*., 2009). While decreasing MZ size through earlier differentiation (Dello Ioio *et al*., 2007), cytokinin affects root length in *Arabidopsis* only at high concentrations (Růžička *et al*., 2009). In the case of *Azolla*, CK did not alter root growth but it did alter MZ cell number. This might be explained by a CK‐induced delay in differentiation and a simultaneous restraint on cell length.

In summary, IAA influenced both cell elongation (in MZ and TZ) and MZ cell number. This resulted in its significant impact on root length, whereas phenotypes triggered by CK were mainly governed by changes in cells of the MZ and not the elongation in *Azolla* roots. This is further supported by the xylem phenotypes that displayed either a delayed or a premature differentiation of proto‐ and metaxylem. More densely ornamented xylem and orthogonal instead of rhomboid metaxylem cells observed upon IAA treatment might be the simple consequence of a delayed or impaired elongation. All of these are linked to the relative position to the apex and are thus probably a direct cause of longitudinal alterations. Our information is limited with regard to understanding the upstream molecular regulation that elicits these phenotypic alterations. For example, the RNAseq analysis revealed *CYP71A20* to be differently regulated upon auxin treatment in comparison to its *A. thaliana* homologue (Goda *et al*., 2004). This might suggest a possible difference in the upstream perception of auxin in the *Azolla* root, but it is just as likely that auxin and cytokinin signalling acts downstream on very specific factors. *Azolla* is currently not accessible for genetic studies. Nevertheless, based on sequence homology, a set of genes were detected that can be associated with the observed phenotypes induced by the phytohormones; that provide information about the *Azolla* root developmental regulon and identity; and that are valuable candidate genes for future studies on fern root development.

### Regulon of *Azolla* roots

The rigid cell walls of plants pose a challenge when a change in phenotype is required. We found that the most strongly regulated genes in *Azolla* roots were those involved in cell wall modification, a major group of which were the expansins that are involved in virtually all processes during plant cell remodelling and growth (Sampedro & Cosgrove, 2005). Of all expansins, the α‐expansins are thought to be the most gene‐rich group in the common ancestor of embryophytes (Sampedro & Cosgrove, 2005). The same is true for the transcriptome of the *Azolla* root. We did not find many orthologous α‐expansin genes between *A. thaliana* and *A. filiculoides*, but we did find co‐orthologous ones. This suggests that after an initial round of expansin family inflation in the common ancestor of embryophytes, several subsequent rounds of lineage‐specific radiations occurred. Expansins influence cell wall loosening, which is linked to the auxin‐mediated acid‐growth hypothesis (Rayle & Cleland, 1992; Link & Cosgrove, 1998; Catalá *et al*., 2000; Cosgrove, 2000). Yet exogenous auxin is known to reduce root length in *Arabidopsis* through altering the cell elongation (Rahman *et al*., 2007; Růžička *et al*., 2009). IAA‐induced reduction in root length and down‐regulation of expansins suggest that the same occurs in *Azolla* roots. Cell wall modification and cellulose deposition are further mediated through microtubule organization (Wasteneys, 2004). Intriguingly, both CK and IAA lead to similar changes with regard to xylem phenotypes and up‐regulation of MOR1 homologues. Owing to the direct link between xylem phenotype and microtubule organization (Oda & Fukuda, 2012), both phenotypes could be explained through the same mechanism: genetically controlled cell wall modification that shapes the *Azolla* root.

Fern root systems are governed by adventitiously arising roots (primary homorhizy); their primary RAM and resulting primary root are only short‐lived (Hou & Blancaflor, 2009). This stands in clear contrast to (secondary) homorhizy in angiosperms, where the primary root is long‐living. Yet, as in angiosperms, each of these adventitious roots has, similar to lateral roots (De Smet *et al*., 2006), its own postembryonic apical meristem in form of a RAC. Regulators of secondarily formed meristems (De Smet *et al*., 2006) are thus a likely component of the *Azolla* root's meristematic signature, not least because the control of adventitious and lateral root formation seems to largely overlap (Gutierrez *et al*., 2009; Bellini *et al*., 2014). Indeed, although *Azolla* does not have any lateral roots, a salient amount of differentially expressed *Azolla* root genes were homologous to lateral root developmental regulators. In angiosperms the determination of the lateral root founder cell identity is largely regulated by auxin (Fukaki *et al*., 2005). Yet, in *Ceratopteris*, auxin affects only the adventitious parent root, but not lateral root development (Hou & Hill, 2002; Hou *et al*., 2004). Consistent with the canonical *Arabidopsis* auxin signalling pathway (Liscum & Reed, 2002), expression of b‐group Aux/IAAs was induced upon IAA, but apparently did not promote the activity of the adventitiously formed RAC in a manner that would result in a larger MZ. Yet, cytokinin can also regulate lateral root development. Homozygous *Arabidopsis wol* mutants display no lateral root emergence from the primary root, but bear only hypocotyl‐borne roots (Scheres *et al*., 1995). We found *WOL* to be one of the most ubiquitous cytokinin signalling components in the *Azolla* root. In addition, a possible homologue of *BREVIS RADIS* (AzfiRT34406; *e*‐value 1.01 × 10^−06^; Table S5) was also detected, which is thought to be crucial for cytokinin's regulation of lateral root formation (Li *et al*., 2008). What could this imply for the evolutionary origin of the euphyllophyte root?

### From thallus to cormus and from adventious to primary roots

Apical growth took place in the common ancestor of all land plants and precedes the evolution of roots (Graham *et al*., 2000; Prigge & Bezanilla, 2010; Pires & Dolan, 2012). Even the most basal plants, including some streptophyte algae (Kenrick & Crane, 1997; Wodniok *et al*., 2011), develop through the action of an apical cell (Graham *et al*., 2000). Liverworts, or hornworts, are often discussed to be the most ancient land plants (Gensel, 2008; Wickett *et al*., 2014) and their thalli grow through marginal meristems (Hagemann, 1999), but also by the activity of apical cells (Goffinet & Buck, 2013). Both sporophytes and gametophytes of ferns (and mosses) bear apical cells mediating their growth (Hagemann, 1999; Prigge & Bezanilla, 2010; Schneider, 2013). Although the roots of lycophytes and euphyllophytes most probably stem from two independent origins, both develop through an RAC (Prigge & Bezanilla, 2010; Pires & Dolan, 2012). Sanders & Langdale (2013) found that auxin and cytokinin act antagonistically on branching in the lycophyte *Selaginella kraussiana*; in that case, auxin led to a higher degree of root branching. It was concluded that this mechanism could be homologous to lateral root development of angiosperms, in which auxin promotes lateral root formation. In the fern *Ceratopteris*, however, lateral root development was not influenced by auxin (Hou *et al*., 2004). This means that while the antagonism of auxin and cytokinin is conserved in lycophytes and euphyllophytes, its outcome for the respective organs is not.

### A shoot‐like origin for euphyllophyte roots?

Fossil records show that early vascular plant roots are strikingly similar to shoots. Roots were therefore predicted to have evolved through dichotomous branching from shoots (Graham *et al*., 2000; Jiang & Feldman, 2005). If we now recall that moss gametophytes and sporophytes share developmental regulators (Menand *et al*., 2007; Bennett *et al*., 2014; Viaene *et al*., 2014), it seems plausible that for the development of the basal fern‐sporophytic body, regulators also found in moss gametophore development were recruited. This process would have then occurred in shoot‐like structures governing a shoot‐first morphology. It has been hypothesized that the RAM arose from the SAM (Jiang & Feldman, 2005). Yet, plant development is often governed by the same regulators, which can act in very different tissues, leading to different outcomes (Prigge *et al*., 2005). We detected contigs with homologies to *Arabidopsis* genes that could be characteristic for either root or shoot. Whether these indicate a more root‐like or shoot‐like regulation of the *Azolla* RAC regulon cannot be stated, not least because of the similarity in RAM and SAM regulation (Stahl & Simon, 2010). Only genetic studies will be able to elucidate the detailed framework that regulates fern RAMs.

The observed phytohormone effect, however, provides intriguing implications for the evolution of root identity. Cytokinin is known as a growth inductor of shoot branches, while auxin inhibits this process (Müller & Leyser, 2011). Both shoot branches and adventitious roots arise laterally from the shoot during postembryonic development. If we entertain the idea of a shoot‐first origin of roots, there are two lessons to be learned from *Azolla* root development with regard to the basal sporophyte root system in euphyllophytes: their origin is adventitious and postembryonic, and they branch off as an altered shoot supported by cytokinin. Whether these adventitious roots are homologous to the embryonic primary roots of seed plants remains to be proven. Yet, it is clear that auxin's role in the dominant organs of the *Arabidopsis* root system does not apply in the same way to the fern *Azolla*. Our data indicate that it might be cytokinin instead. The key will be to determine the identity of the transiently present RAM of the fern embryo. It further raises the question at what point during euphyllophyte evolution the first permanent embryonic roots emerged, and whether this process was the advent of auxin's dominance during (lateral) root developmental regulation.

## Author contributions

J.d.V and S.B.G. planned and designed the research. A.M.F. and J.d.V. performed experiments. J.d.V., M.R. and S.R. performed bioinformatic analyses. All authors discussed the results and commented on the manuscript that J.d.V. and S.B.G drafted. The final version of the manuscript was written by J.d.V., H.S., A.B., A.C. and S.B.G. and approved by all authors .

## Supporting information

Please note: Wiley Blackwell are not responsible for the content or functionality of any supporting information supplied by the authors. Any queries (other than missing material) should be directed to the *New Phytologist* Central Office.

**Fig. S1** Root hair development in *Azolla*.
**Fig. S2** *Amyloplasts* line the *Azolla* stele.
**Fig. S3** Regeneration of the meristematic zone.
**Fig. S4** Conformation of global gene expression trends through two‐step quantitative reverse‐transcription PCR.
**Fig. S5** Ranked gene ontology analysis of global gene expression in *Azolla* roots.
**Fig. S6** Evolution of expansins.
**Fig. S7** Phylogenetic analysis of *Arabidopsis* and regulated *Azolla* Aux/IAA proteins.

**Table S1** Overview of the RNAseq analysis
**Table S2** Annotations and expression values of the *Azolla filiculoides* root tip transcriptome
**Table S3** Oligonucleotides used for two‐step qRT‐PCR
**Table S4** Sequences used for the alignment of land plant expansins
**Table S5** Known developmental regulators expressed in *Azolla* root tips
**Table S6** Significantly regulated contigs upon auxin and cytokinin treatment and GOterm enrichment analysis depicted in Figs 5 and 7
