# Supplementary material for: Cytokinin‐induced promotion of root meristem size in the fern Azolla supports a shoot‐like origin of euphyllophyte roots
Source: New Phytol. 2015 Sep 11;209(2):705–20. doi: 10.1111/nph.13630 (PMC5049668; doi:10.1111/nph.13630)
Supplement: Supplementary file 1 — Fig. S1 Root hair development in Azolla. Fig. S2 Amyloplasts line the Azolla stele. Fig. S3 Regeneration of the meristematic zone. Fig. S4 Conformation of global gene expression trends through two‐step quantitative reverse‐transcription PCR. Fig. S5 Ranked gene ontology analysis of global gene expression in Azolla roots. Fig. S6 Evolution of expansins. Fig. S7 Phylogenetic analysis of Arabidopsis and regulated Azolla Aux/IAA proteins. [file NPH-209-705-s001.pdf]

Supporting Information Figs S1–S7

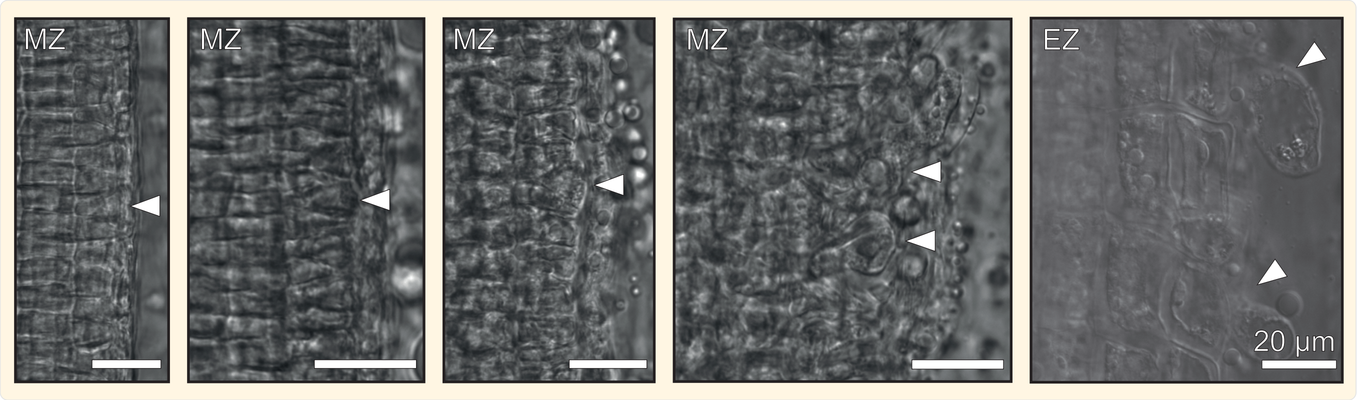

Figure S1. Root hair development in *Azolla*. Along the *Azolla* root, cells that will give rise to root hairs (arrow heads) become distinguishable already in the meristematic zone (MZ) and fully develop in the elongation zone (EZ).

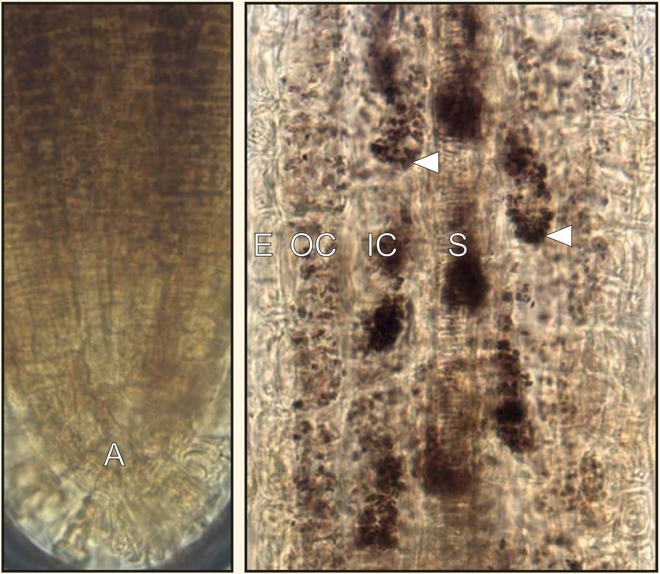

Figure S2. Amyloplasts line the *Azolla* stele. *Azolla* roots stained with 5% Lugol's iodine reveal several amyloplasts (arrowheads) lining the stele above the meristematic zone (right) and accumulating rootwards, but are absent from the root apex (left). Epidermis (E); Outer Cortex (OC); Innter Cortex (IC); Stele (S); Apical cell (A).

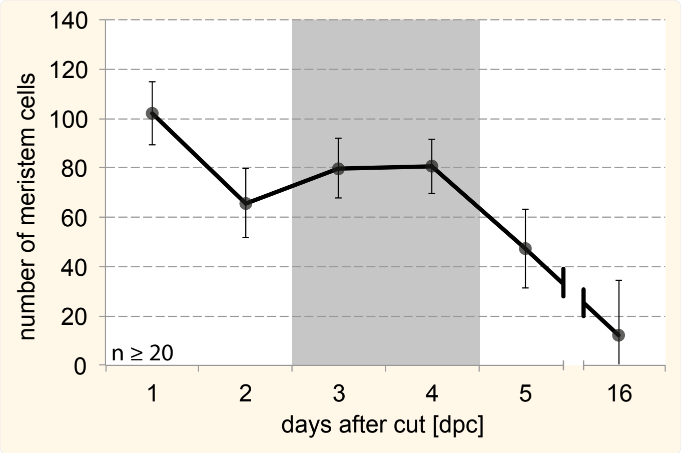

Figure S3. Regeneration of the meristematic zone. Graph depicting the number of meristematic cells in newly forming roots one to sixteen days after complete removal of the old roots (days post cut; dpc) from the sporophyte body. For each data point more than 20 roots were analysed; Error bars depict the standard deviation. Note that after 16 days most root meristems had terminated.

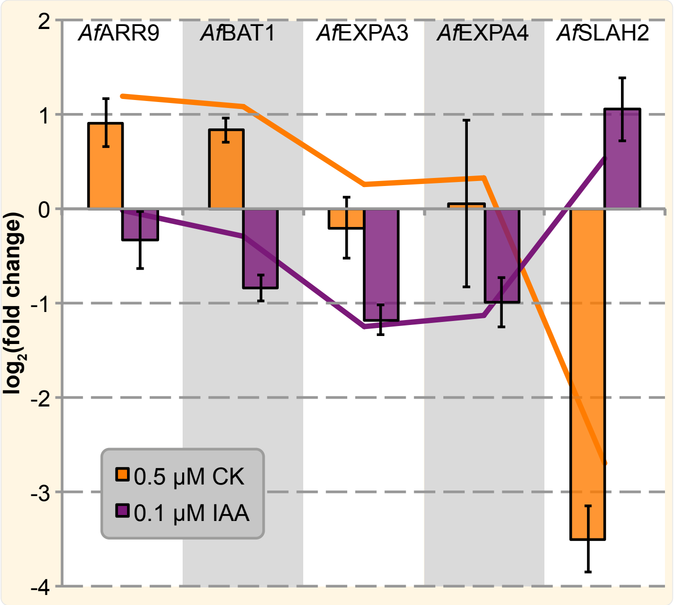

Figure S4. Conformation of global gene expression trends through two-step quantitative reverse-transcription PCR. Relative expression of five genes were analysed using two-step quantitative reverse transcription PCR (qRT-PCR; histogram) to confirm the global gene expression trends analysed via RNAseq (graph). All expression data is shown as a log2(fold change). Two-step qRT-PCR data was retrieved from biological triplicates and ΔΔct-normalized to mock treatment and using Af*CAM5*(AzfiRT00154) and Af*TUA2*(AzfiRT00021) as reference genes. Error bars indicate standard deviation for the biological triplicates.

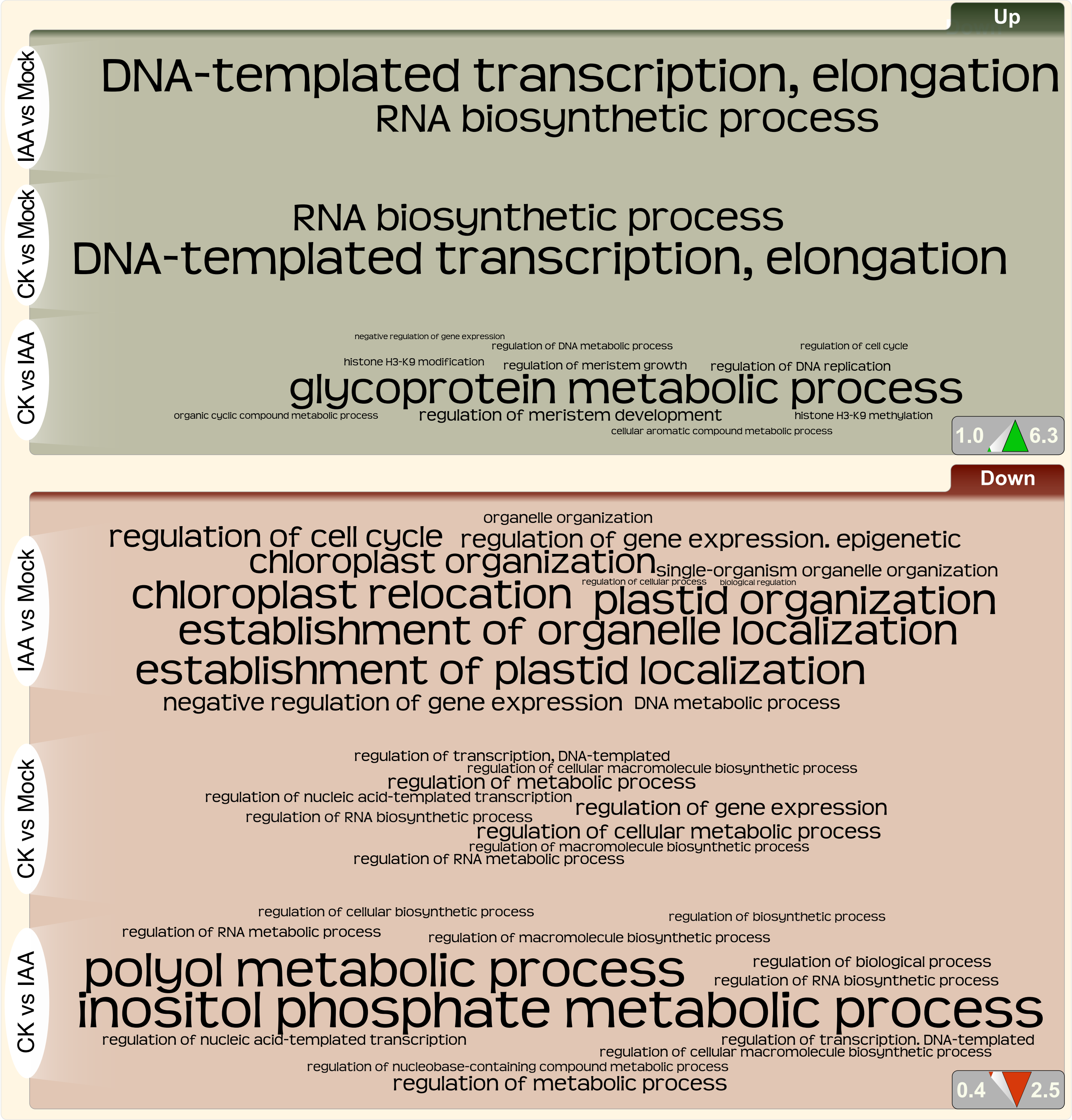

Figure S5. Ranked Gene Ontology analysis of global gene expression in *Azolla* roots. Gene ontology (GO) enrichment analysis (p<10-3) based on the *Arabidopsis* annotation (e value<10-7) of a ranked list of the expressed genes in roots comparing 0.1 µM IAA vs. mock, 0.5 µM CK vs. mock and 0.5 µM CK vs. 0.1 µM IAA treatments. GO-term enrichment based ranked upregulation is shown on the top, downregulation is shown at the bottom. Sizes of the GO-terms are based on log2(enrichment), see key in right corner.

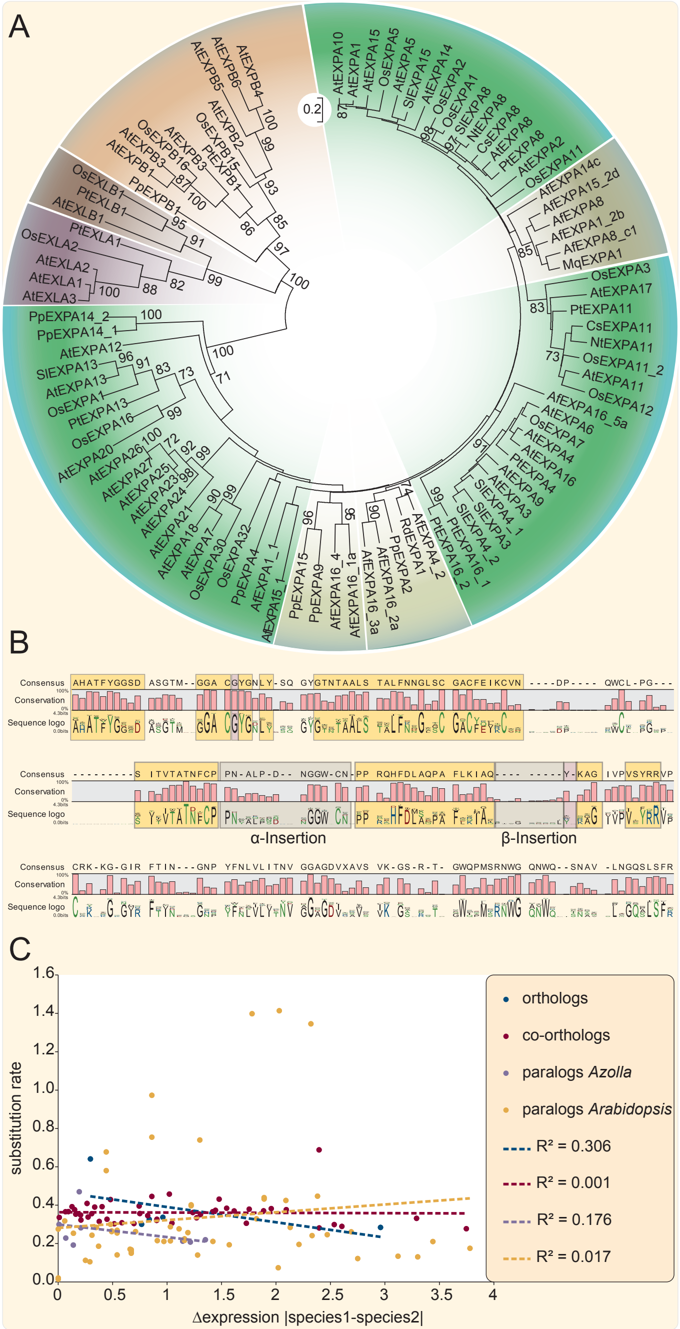

Figure S6. Evolution of expansins.

(A) Phylogenetic analyses of the expansin family. The maximum likelihood-based phylogeny shows a clear distingtion of four major expansin subfamilies (a): α-expanins (green), α-like expansins (purple), β-expansins (orange) and β-like expansins (brown). Bootstrap values >70 are given on the branches. Most of the expansin orthologous sequences retrieved from the *Azolla* root transcriptome were α-expansins. γ-expansins were not included due to absence in the transcriptome. Within the α-expanins one strongly supported clade consisting of only fern-expansin orthologs and two strongly supported clades containing only fern and moss expansin-orthologs are formed (light green). These three clades contain the majority of the potential orthologous α-expansin sequences retreived from the *Azolla* transcriptome. A list of the used sequences can be found in Table S6.

(B) Conservation of expansin sequences. The consensus sequences of the sequence alignment (Table S6) used for the phylogenetic analyses is given above the conservation signal of those consensus amino acids (ranging from 0-100%). The sequence logo below shows the relative distribution of amino acids per postion. Highly conserved areas are indicated by yellow (previously published) or purple (new) boxes. Grey boxes indicate the expansin-type specific insertions that only occur in either α-expanin or β-expansin sequences.

(C) The pairwise substitution rate between orthologous (blue), co-orthologous (red), paralogous (purple and yellow) sequences from *Azolla* and *Arabidopsis* expansin sequences is plotted against the absolute pairwise difference of expression (in log_2[fold change] of Auxin vs. Mock) between the two compared sequences in the *Azolla* and *Arabidopsis* root. Phylogenetic distance does not correlate with expressional differences of the expansins.

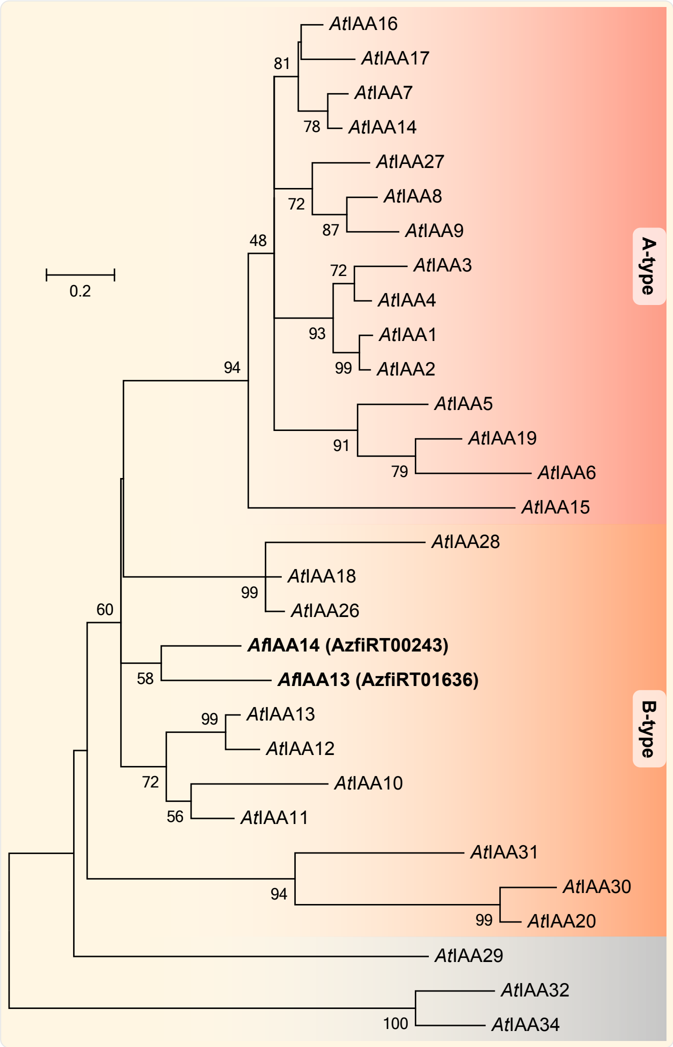

Figure S7. Phylogenetic analysis of *Arabidopsis* and regulated *Azolla* Aux/IAA proteins. Protein data was extracted from the longest ORF found in the significantly regulated *Azolla filiculoides* Aux/IAA homologs and aligned with *Arabidopsis* Aux/IAA proteins (extracted from TAIR) using MAFFT G-INS-I. A maximum-likelihood (ML) tree was computed using 1000 bootstrap replicates and a JTT+G substitution model with 5 gamma categories and a partial deletion cutoff of 95%. Bootstrap values <50 are not shown. The best-fit substitution model was determined using MEGA. A- and B-types were classified according to Remington *et al.,* 2004.

Remington DL, Vision TJ, Guilfoyle TJ, Reed JW. 2004. Contrasting modes of diversification in the *Aux/IAA* and *ARF* gene families. *Plant Physiology* **135:** 1738-1752.
